# On the Relationship between Mechanical Properties and Crystallisation of Chemically Post-Processed Additive Manufactured Polylactic Acid Pieces

**DOI:** 10.3390/polym12040941

**Published:** 2020-04-18

**Authors:** A.P. Valerga, S.R. Fernandez-Vidal, F. Girot, A.J. Gamez

**Affiliations:** 1 Department of Mechanical Engineering and Industrial Design, School of Engineering, University of Cadiz, Av. Universidad de Cádiz, 10, E-11519 Puerto Real, Cadiz, Spain; raul.fernandez@uca.es; 2 IKERBASQUE, Basque Foundation for Science, 48013 Bilbao, Spain; franck.girot@ehu.eus; 3 Faculty of Engineering, University of the Basque Country, Alameda de Urquijo s/n, 48013 Bilbao, Spain; 4 Mathematical Engineering Research Group, School of Engineering, University of Cadiz, Av. Universidad de Cádiz, 10, E-11519 Puerto Real, Cadiz, Spain; antoniojuan.gamez@uca.es

**Keywords:** manufacturing design, hardness, tensile strength, crystallite, polylactic acid, fused deposition modelling, finishing processes, biodegradable polymer

## Abstract

Nowadays, improvement of the surface finish of parts manufactured by fused deposition modelling is a well-studied topic. Chemical post-treatments have proven to be the best technique in terms of time consumption and smoothness improvement. However, these treatments modify the structure of the material and, consequently, its mechanical properties. This relationship was studied in this work. In this case, on the basis of a previous study on crystallisation, polylactic acid pieces were subjected to different post-treatments to evaluate their effects on the sample’s mechanical properties, i.e., tensile strength and hardness. Models were obtained according to their percentage of crystallisation, which was related to the different treatments, as well as immersion time. Dramatic changes were obtained within a wide range of material behaviour with some treatments. Specifically, changes were obtained in the maximum stress (from 55 to 20 MPa), in elongation (from 3% to 260%), and in the hardness scale (Shore D to A).

## 1. Introduction

The eco-sustainability of manufacturing processes is a fundamental issue considering its potential for energy savings, material savings and life extension, recycling and process optimisation [1]. Additive manufacturing processes and, in particular, fused deposition modelling (FDM), improve this aspect more than other conventional manufacturing technologies. However, the use of petrochemical-based plastics is becoming increasingly limited. For this reason, the study of biodegradable materials, in particular, polylactic acid (PLA), is proposed as an alternative so that this process can be transferred to a bio-sustainable industry [2].

Despite being a widely used polymer in industry [3], PLA in FDM processes produces a poor surface finish and low dimensional accuracy. This seems to be the main obstacle to the commercial production of this material through this process. Nevertheless, many researchers are already studying the improvement of the surface finish by means of pre- and post-processing techniques on parts manufactured by FDM [4]. Some authors try to improve the surface quality through the selection of adequate process parameters (pre-processing techniques), but the improvements are usually equal to or less than 10% [5]. According to a recent analysis of the subject, the most pronounced improvements in surface quality are usually related to post-processing techniques [6].

Many methods have been proposed for the permanent surface modifications of PLA pieces, such as alkaline surface hydrolysis, atom transfer polymerisation, photografting by UV light, plasma treatment or chemical reactions after plasma treatment [6]. These processes are carried out to achieve certain special characteristics, not to specifically improve the surface finish. According to the research literature, the technique used to further reduce roughness is chemical post-processing, either by vapour smoothing or by immersion in liquids.

For example, R. Singh et al. achieved up to 99% improvement in the surface quality of FDM parts through a 24-hour vapour smoothing process [7]. A. Lalehpour et al. managed to significantly improve the roughness of ABS (Acrylonitrile butadiene styrene) samples by up to 95% using shorter times (between two and eight cycles of 15 seconds) [8]. In another study, A. Garg et al. used cold vapour, obtaining a less significant improvement, and analysed other aspects, such as the dimensional deviations that these treatments caused in the parts [9]. Finally, our group achieved improvements in the roughness of up to 97% with rapid immersion (less than a minute) in organic solvents [10].

However, few of these works have analysed the impact on the material after the application of these treatments. As shown in some publications, the mechanical properties of the parts are highly affected by the application of a chemical product, but most of these publications do not explain the change in material behaviour.

A. Garg et al. studied the small reduction (only 5%) in the mechanical tensile and flexural strengths of ABS samples subjected to cold dimethyl ketone vapours [11]. Y. Jin et al. studied PLA with chemical post-processing and obtained not only improvements in the surface finish but also a 63% reduction in tensile strength and a 50% higher elongation at break than the untreated polymer [12].

In summary, neither an in-depth study of the change in the mechanical behaviour of PLA pieces after chemical treatments has been carried out, nor has it been analysed in terms of the change in the structure of the material. For example, some PLA baths subjected to different immersion treatments have shown partial crystallisation [10]. Thus, it is necessary to understand the effect of the treatment on the mechanical properties in order to control them. Consequently, this work aims to study the mechanical behaviour, specifically tensile strength and hardness, of parts that are immersed in the same previously studied solvents to complete the research based on the partial crystallisation of the material. The solvent treatments studied in this work (ethyl acetate (C_4_H_8_O_2_), tetrahydrofuran (C_4_H_8_O), dichloromethane (CH_2_Cl_2_) and chloroform (CHCl_3_) do not significantly alter the chemical nature of the PLA polymer, although they do lead to changes in the microstructure of this material and thus in the mechanical and thermal properties of the parts. Therefore, a direct relation between crystallisation and the indicated mechanical properties is proposed.

## 2. Materials and Methods 

Polylactic acid (PLA) from FFF World was used in this work. Three different colours were initially analysed (white, grey and black), and natural (colourless) PLA was chosen for the rest of the experiments.

**Figure 1:**
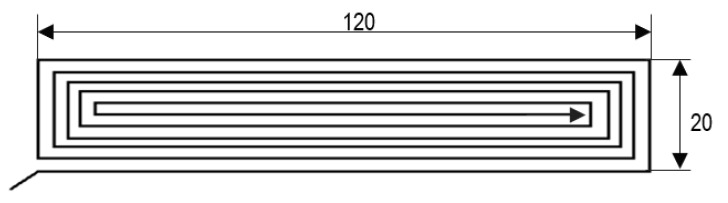
Dimensions and trajectories used for monolayer samples.

A 0.4-mm-diameter nozzle was used on a test bench to manufacture the samples. Monolayer specimens were manufactured for tensile strength tests, and hexahedral specimens were used for hardness tests. For monolayers, different possible trajectories were analysed, as in S-H. Anh et al. [13] or C. Wendt et al. [14]. The best specific geometries for single-layer samples and their dimensions and trajectories for carrying out studies with tensile tests are shown in Figure 1. The monolayer thickness was 0.8 mm. On the other hand, the hexahedral samples were prismatic specimens with a square base with 30-mm sides and a 5-mm thickness. For these last samples, infill was 100% rectilinear, with a 0° raster orientation and three top and bottom layers. The layer thickness of these specimens was 0.2 mm.

The most important manufacturing parameters used are shown in Table 1. Once monolayers were manufactured, they were immersed in four organic solvents: ethyl acetate (C_4_H_8_O_2_), tetrahydrofuran (C_4_H_8_O), dichloromethane (CH_2_Cl_2_) and chloroform (CHCl_3_). The immersion times (*t*_i_) for each solvent were 1, 30 and 60 s. Hexahedral samples were made for hardness tests. Only the natural material was analysed and immersed in the four aforementioned solvents for a period of 60 s.

Five test parts were manufactured for each experiment, as stated in the standard. Some of these solvents have been used in other studies and have shown favourable behaviour towards the improvement of the surface quality of the parts manufactured with FDM. Other studies have demonstrated partial crystallisation (*c*) of the material after the indicated treatments and conditions (Table 2) [10]. The approximate crystallisation percentage was estimated by image analysis from XRD and DSC diagrams. The area enclosed under the shoulder of a phase, contrasted with the crystalline peak of that same phase, was computed. This percentage of crystallisation is intended to be related to the mechanical tests explained below.

A universal testing machine Shimadzu^®^ AG-X (Shimadzu, Kyoto, Japan) was used for the mechanical tests. These tensile tests were performed with a continuous testing speed of 2 mm/s, as recommended in standards for moulding and extrusion polymers [15]. These tests were carried out under temperature and humidity conditions similar to those of service parts (25 °C and 50%).

Some treated samples displayed high elongation. For this reason, tests were planned at the initially proposed speed (2 mm/s) and also at a speed that was 10 times higher (20 mm/s) but within the range indicated in the standard.

Shore-D-type hardness measurements, suitable for hard rubbers and thermoplastic, were made according to the recommendations of the current Additive Manufacturing standard [16] with a portable HPE instrument and a Bareiss^®^ BS61 holder (Bareiss Heinrich Prüfgerätebau GmbH, Oberdischingen, Germany). To carry out the tests, the recommendations established in the UNE-EN ISO 868 standard were followed [17]. Five hardness measurements were taken per sample for 15 seconds and separated from each other and from the edges of the piece by at least 9 mm in all directions.

## 3. Results and Discussion

**Figure 2:**
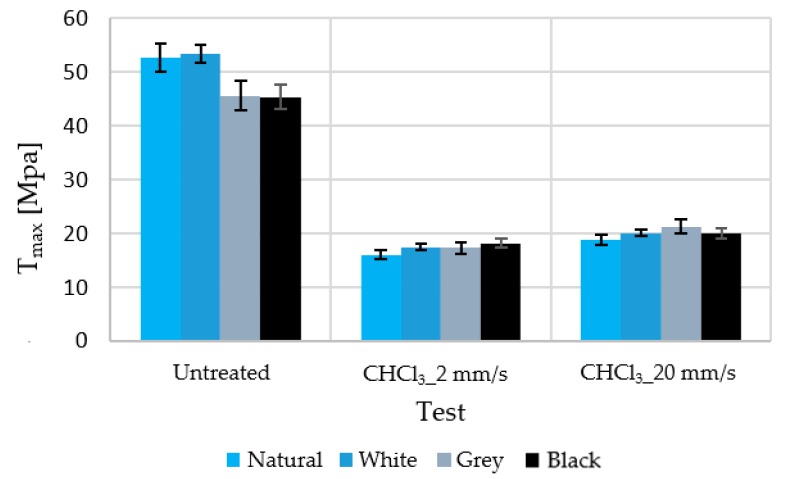
Ultimate tensile strength of treated and untreated samples tested at two test speeds.

Figure 2 shows the maximum stress supported by untreated and chloroform-treated samples at two different test speeds.

As mentioned before, two test speeds were considered. A slight dependence of the test speed on the maximum stress and elongation was observed. This is due to the occurrence of deformation hardening. After the start of plastic deformation, an additional flow continues at higher stress. This is probably caused by a decrease in the free volume in the shear bands, which appear after the beginning of the plastic flow. This causes a slight increase in the maximum stress and a slight decrease in the elongation [18]. However, with this change in speed, the results varied in less than 10% (with the dispersion at 5%); owing to the test time and volume of data obtained, a test speed of 20 mm/s was used for all cases in which deformations of more than 50% occur.

Although the manufacturer claims that only 1% of this material is additive (colour and stabiliser), the observed changes were not the same for all colours. However, little difference was observed between coloured samples that were subjected to immersion in solvents, despite the existence of a more substantial difference prior to these treatments.

The arrangement of the chains should homogenise the load applied to the polymer, and therefore, a higher degree of crystallinity should show greater tensile behaviour. Nevertheless, this statement is not supported by the experiments. For this reason, it is plausible that fractures could have occurred by the unravelling of the chain, where the unbroken molecules are separated from each other, because the probability of chain disentanglement is dependent on the length of the molecules and the degree to which they are intertwined [19]. 

**Figure 3:**
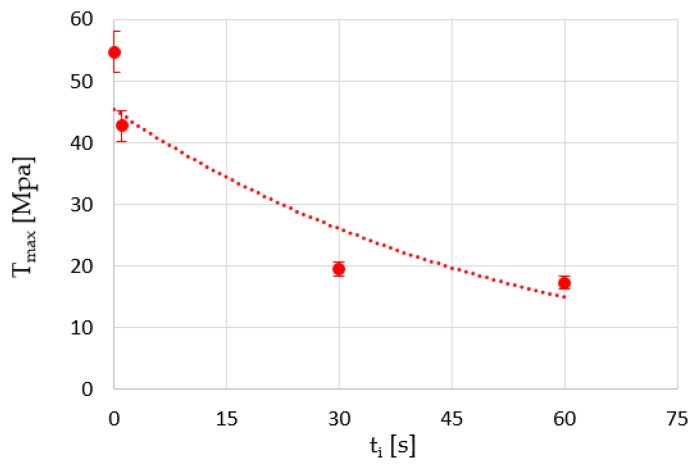
Ultimate tensile strength of chloroform-treated samples at different immersion times.

On the other hand, it is estimated that the immersion time (*t*_i_) is a significant variable in the results. Therefore, its influence on the tensile strength of samples that were subjected to chloroform treatment was studied (Figure 3).

The results suggest that an extended immersion time reduces the maximum voltage value to a maximum that seems not too far from the range set for that material. This means that the stress obtained after a chloroform treatment can be assumed to be at least about 17 MPa, which means a reduction of about 68% in the maximum stress of the material.

The polymer appears to exhibit shear or cracking deformation. This means that the molecules of the polymer slip with respect to each other when subjected to stress. Since the polymer has been partially arranged (partial crystallisation), the deformation is highly localised and leads to cavitation or vacuum formation, where the chains form aligned packages or fibrils [19].

**Figure 4:**
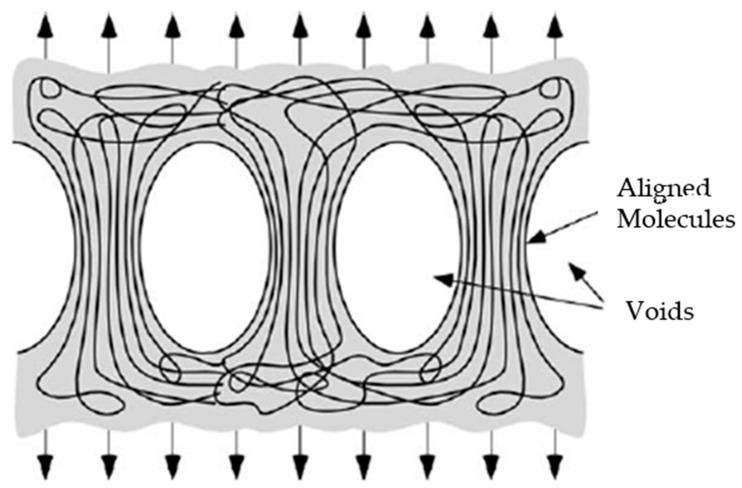
Voids form between fibrils, which are bundles of aligned molecular chains.

**Figure 5:**
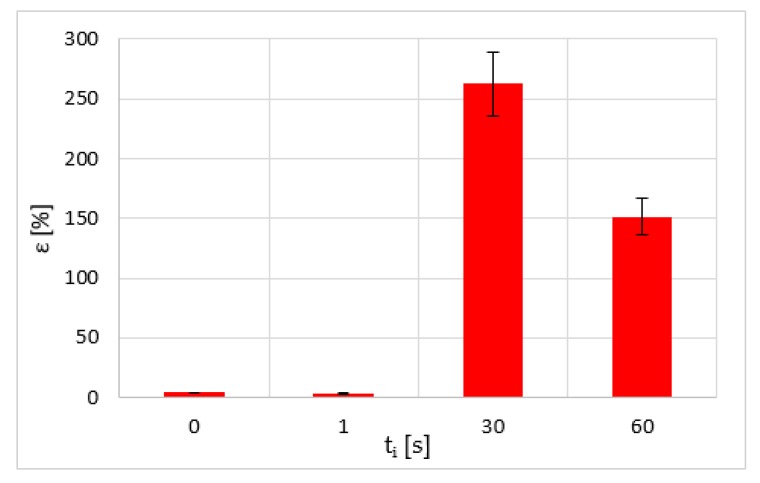
Elongation at break of chloroform-treated samples at different immersion times.

Existing voids expand and new voids appear as a result of incompatibility between fibrils, accompanied by the expansion of the solvent inside these pores (Figure 4). As the structure becomes more aligned, these fibrils support more unbalanced stresses with respect to the amorphous structure zones and therefore have a greater capacity for deformation than the untreated parts (Figure 5). Fracturing occurs when some of these fibrils break and the stress distributed to adjacent ones is sufficient to completely separate the material in that area. However, there seems to be a period of immersion (about 30 s) after which the polymer saturates. Thus, longer exposure to the solvent increases the absorption of the polymer and the expansion of the solvent to a greater extent. This translates into a higher concentration of cavities formed or expanded by the trapped molecules (in this case, chloroform), causing more premature material failure.

The craze zone grows by drawing additional material into the fibrils. Adapted from Anderson [19].

**Figure 6:**
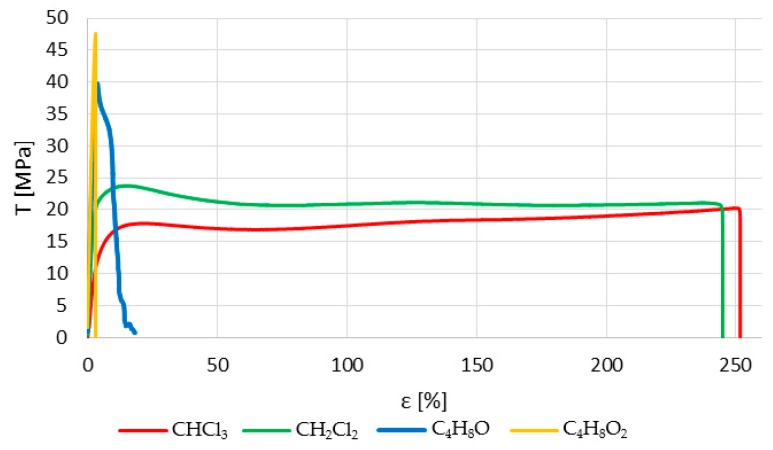
Typical experimental stress–strain curves of the differently treated samples.

Figure 6 shows representative stress–strain curves of samples immersed in different solvents for a 30-second immersion period. On the one hand, halogenated or chlorinated solvents provide important ductility to the plastic and, thus, an elastoplastic zone whose limit is close to the maximum stress supported. In this way, these materials can be used for low-load applications where some recovery is needed. On the other hand, non-halogenated solvents do not modify the fragility of the polymer in such a transcendental way, although higher ductility is achieved than in the untreated samples. However, in all cases, the ultimate tensile strength supported by the polymer is lower after being treated with any of the solvents.

**Figure 7:**
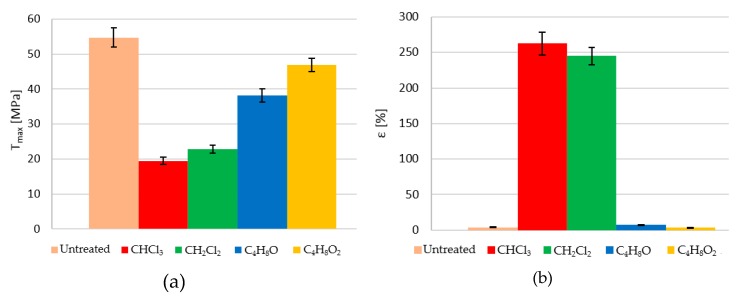
(**a**) Ultimate tensile strength and (**b**) elongation at break of different treated samples.

Figure 7a shows the average maximum stresses obtained, while Figure 7b shows the average maximum deformation obtained in each case. A direct relationship between load and fragility can be seen, with hardly any elastic zone and less elongation.

**Figure 8:**
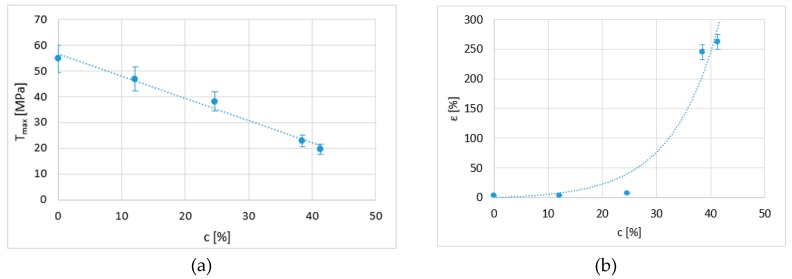
(**a**) Ultimate tensile strength and (**b**) elongation at break as a function of the percentage of crystallisation of the samples.

A relationship of direct dependence was observed between the tensile behaviour of the samples and their percentage of crystallisation. In this respect, the maximum stress and deformation were calculated as a function of this percentage of crystallisation. Figure 8a shows the maximum stress as a function of the percentage of crystallisation obtained from each applied chemical treatment. The graph presents a linear trend that shows that a higher percentage of crystallisation results in a lower resistance. Additionally, when the percentage of crystallisation increases to over 25%, elongation increases exponentially (Figure 8b).

In addition, a limit is detected at which the material becomes as plastic as possible. According to the literature [20,21], these limits are a minimum stress of 14 MPa and a maximum elongation at fracture of 400%. These results are not very far from the ones obtained in this study. This relationship with crystallisation is approximate, and although the confidence value is acceptable, future studies should consider other aspects, such as the size of the domains formed in each case and the phases of the polymer present in each sample. 

To our knowledge, there are no studies that relate the tensile strength (*T_max_*) supported by PLA FDM manufactured parts to their elongation at break (ε) and to the subsequent application of different solvents used to improve the surface quality. 

For this reason, an attempt can be made to find a mathematical model to obtain the approximate responses that define the tensile behaviour according to one of the variables used, partial crystallisation of the sample (*c*), as this seems to be a very significant variable in the results.

The following fitting equations are proposed:

(1) $$T_{max}=A+B\cdot c$$

(2) $$\varepsilon =A\cdot \left(e^{B\cdot c}-1\right)$$

Table 3 includes the values of these coefficients in order to obtain the *T_max_* and ε responses as the most representative parameters of the tensile behaviour. The correlation coefficient (*R*) for each equation shows the accuracy of the fitting. 

Preliminary tests showed that PLA has a Shore D hardness that corresponds to thermoplastics and hard rubbers. However, the hardness value drops considerably when certain treatments are performed, entering the upper limits of the A scale, which is used to test materials classified as elastomers, plastics and soft rubbers. In order to make a better comparison, all tests were carried out under the same conditions, maintaining the hardness scale. 

The direct dependence on the type of treatment is mainly associated with the degree of crystallinity provided by each one, so hardness decreases with crystallinity. The arrangement of chains or formation of crystals leaves volumes of material with unravelling of the molecules and more free zones. Because the crystals are so small, they leave a proportionally large area of voids that diminish the mechanical properties of the material [22]. This accounts for the variation in hardness values.

In order to carry out a more precise evaluation of the mechanical properties, factors such as the size of the domains formed in each case, the presence of different phases or even the appearance of micropores should be taken into account. A more in-depth study will be undertaken in this regard.

**Figure 9:**
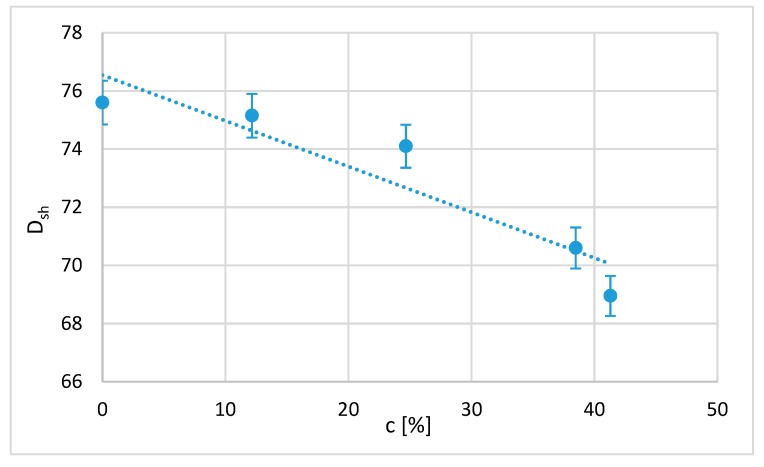
Shore D hardness as a function of the crystallinity [%] obtained from each treatment.

As expected, since hardness is directly related to the stress–strain graphs, parametric models of Shore D hardness can be established as a function of PLA crystallisation (Figure 9). In this case, a linear relationship is enough to correlate both variables. It is expected that considering more variables, such as the time of immersion and the size of the domains or the phases present in the sample, will show a more complicated dependence. Thus, the D-type Shore hardness (*D*_sh_) obtained as a function of the percentage of crystallisation (*c*) is

(3) $$D_{sh}=76.5-0.157c$$

The correlation coefficient R = 0.87, which shows that the dependence of the mechanical behaviour of the polymer on the crystallisation percentage is very important. This result has not been found in previous works.

## 4. Conclusions

PLA parts exposed to chemical solvents undergo partial crystallisation, and the highest percentages of crystallisation are obtained with halogenated solvents. However, the size of the crystalline domains is similar for all the cases studied. Partial crystallisation enhances the thermal stability and resistance of the parts, which can be very useful for certain applications, such as temperature changes. This method could be used to replace parts that are currently made with ABS and help the environment. These treatments have previously been related to the improvement of the surface finish. However, until now, there have been no studies that related the modification of the structure of the material to its mechanical properties.

All organic solvent immersion treatments studied in this work result in a loss of tensile strength and an increase in the elongation at break of the polymer. Thus, halogenated solvents transform natural, brittle PLA into a more ductile polymer with a considerable elastic range, while non-halogenated solvents do not modify this property in a significant way. With halogenated solvents, the stress of the original PLA decreases by more than 50%, providing a deformation of approximately 250%, while the change is not as accentuated in non-halogenated solvents. In addition, an exponential influence of the exposure or immersion time in the chloroform bath is shown, which is assumed to be similar for the remaining treatments. All observed phenomena are associated with the partial crystallisation of the samples subjected to these treatments, and therefore, parametric models that depend on this variable are proposed. Nevertheless, according to the available data, the dependence on other parameters related to crystallisation should be studied. This could reveal the variables that have a significant impact on mechanical properties, such as the size of the crystalline domain, which has proven to be very similar in the studies that have been carried out. 

Furthermore, analysis of the data obtained from the hardness of the material reveals that there are variations in scale that could be transferred to very different applications, depending on the chemical bath to which the part in question is exposed. In this way, as with the previous mechanical properties, a direct dependence between the hardness and the degree of crystallinity of the polymer is observed, with a decrease in hardness associated with an increase in percentage of crystallinity.

## Figures and Tables

**Table 1:** Values of manufacturing parameters.

	Speed (mm/s)	Overlap (%)	Nozzle Temperature (°C)	Bed Temperature (°C)	Retraction (mm and mm/s)
**Value**	20	55	220	65	1.7 and 15

**Table 2:** Values of percentage of crystallised samples.

	Natural	C_4_H_8_O_2_	C_4_H_8_O	CH_2_Cl_2_	CH_3_Cl_3_
**Crystallisation, *c* (%)**	≈0	12.15	24.60	38.50	41.70

**Table 3:** Constants and correlation coefficients obtained for the traction model.

	A	B	R
*T_max_*	56.50	−0.86	0.98
ε	2.57	11.40	0.91
